# Spatial distribution of phenotypic and biotic traits in cassava fields across Sierra Leone

**DOI:** 10.1007/s44279-026-00798-8

**Published:** 2026-10-20

**Authors:** Vandi Amara, Alusaine Edward Samura, Prince Emmanuel Norman, Seka John Steven, Fidele Tiendrebeogo, Angela Obiageli Eni, Justin S. Pita

**Affiliations:** 1 Department of Crop Protection, School of Agriculture and Food Sciences, Njala University, Njala Campus, Freetown, Sierra Leone; 2 Germplasm Enhancement and Seeds System, Sierra Leone Agricultural Research Institute (SLARI), Freetown, Sierra Leone; 3 Regional Center for Excellence for Transboundary Plant Pathogens, Central and West African Virus Epidemiology (WAVE), Pôle Scientifique et d’Innovation de Université Félix Houphouët-Boigny (Bingerville), Abidjan, BPV 34 Côte d’Ivoire

**Keywords:** Trait distribution, Morphological, Disease, Pests, *Manihot esculenta*, Farmers’ fields

## Abstract

Limited information regarding the spatial distribution of key agronomic and biotic traits in cassava morphological types constrains targeted breeding, conservation and crop improvement efforts in Sierra Leone. This study aimed to assess the distribution of key agronomic traits of cassava morphological types on 276 smallholder farms in Sierra Leone during January 2022. Traits studied included apical leaf color, leaf color, leaf vein color, number of lobes, number of whiteflies and grasshoppers, cassava mosaic disease (CMD) severity, farm size and age of plants. A field survey involving 276 cassava farms distributed across the five regions of Sierra Leone was conducted in January 2022. Agronomic, pest and disease data were collected from 30 randomly selected plants per farm following WAVE harmonized protocols. Geographic Information System (GIS)-based geospatial analyses were used to generate spatial distribution maps, while analysis of variance and Pearson correlation analyses were used to examine relationships among traits, pest abundance and disease severity. The majority of the cassava morphological types assessed exhibited green apical leaf (65.3%), light green leaf (64.3%), green leaf vein (55.6%), and prominent foliar scar (53.5%) with seven lobes (62.6%). Whitefly and grasshopper had higher populations of seven whiteflies per plant and 12 grasshoppers per plant in the western and eastern regions, respectively; whereas, a few farms in the southern region had no whiteflies. The CMD infection was mainly through contaminated cuttings (99.2%), with cutting-derived plants exhibiting moderate CMD severity. Mean CMD severity significantly (*P* ≤ 0.05) varied with farm size, with high (4.29), medium (3.8) and low (2.94) CMD recorded in small (0–1 ha), medium (2–3 ha) and big (4–5 ha) farms, respectively. This study establishes the existence of useful phenotypic variability in the regional distribution of morphological traits that could be exploited for phenotypic selection for production, conservation, utilization, and/ or genetic improvement of the crop. Findings serve as useful guide for genotypic selection for production, conservation, utilization, and genetic improvement of cassava using existing phenotypic diversity.

## Introduction

Cassava (*Manihot esculenta* Crantz) is one of the most important staple food crops in tropical and subtropical regions, providing food, income, and livelihood support to millions of households worldwide. The crop is particularly important in sub-Saharan Africa, where it contributes significantly to food security because of its ability to tolerate drought, adapt to diverse agroecological conditions, and produce acceptable yields under low-input farming systems [1, 2]. In Sierra Leone, cassava is cultivated extensively across all regions and serves as a major source of dietary carbohydrates for rural and urban populations. Beyond its importance as a staple food, cassava contributes to household income generation, employment creation, livestock feeding, and agro-industrial development [1]. Recent efforts to improve cassava productivity and resilience have focused on the identification, characterization, and utilization of genotypes possessing desirable agronomic traits, including disease resistance, adaptability, and enhanced production potential [2].

Understanding the spatial distribution of agronomic traits within crop populations is fundamental for effective crop improvement, germplasm conservation, and sustainable agricultural development. Geospatial analytical techniques, particularly those based on Geographic Information Systems (GIS), provide valuable tools for assessing the geographic distribution of crop characteristics, pest populations, and disease incidence across production landscapes [3, 4]. Such approaches facilitate the identification of regions harboring desirable traits, areas vulnerable to pest and disease outbreaks, and environments suitable for targeted varietal deployment and breeding interventions. Geospatial analysis has been widely used to investigate spatial patterns of crop pests and diseases, providing critical information for agricultural planning, surveillance, and management [4, 5]. Furthermore, the integration of GIS technologies with field-based assessments enables researchers to visualize spatial variability, evaluate genotype–environment interactions, and support evidence-based decision-making for crop improvement programs and agricultural policy development [6, 7].

Despite the importance of cassava in Sierra Leone, information on the spatial distribution of agronomic traits, pest abundance, and disease occurrence among cassava morphological types remains limited. Previous studies in the country have largely focused on farmer preferences, varietal selection, agro-morphological characterization, and disease incidence without incorporating geospatial approaches to visualize trait distribution patterns across regions [1, 2]. Existing evidence suggests that considerable morphological diversity exists among cassava morphological types cultivated in Sierra Leone and that environmental conditions may influence the expression and distribution of important agronomic characteristics [2, 7]. However, there is currently a lack of spatially explicit information describing how these traits are distributed across major cassava-growing areas and how they relate to pest abundance, cassava mosaic disease occurrence and the dominant traits preferred by farmers. This knowledge gap constrains efforts aimed at targeted germplasm collection, conservation planning, breeding program development, and strategic deployment of improved varieties. Therefore, this study aimed to assess the spatial distribution of key agronomic traits, pest abundance, and cassava mosaic disease expression among cassava morphological types cultivated across Sierra Leone using geospatial and statistical analytical techniques.

## Materials and methods

### Description of the study area

The study was carried out in five regions of Sierra Leone namely: eastern, northern, northwest, southern and western. The southern region comprises four districts: Bo, Bonthe, Moyamba, and Pujehun. The eastern region comprises of Kono, Kenema and Kailahun districts. The northern region consists of four districts: Bombali, Falaba, Koinadugu, and Tonkolili. The northwest region encompasses three districts: Kambia, Karena, and Port Loko; and the western region comprised western urban and western rural. The geolocation coordinates of the surveyed farm sites were utilized for the generation of a map.

**Fig. 1:**
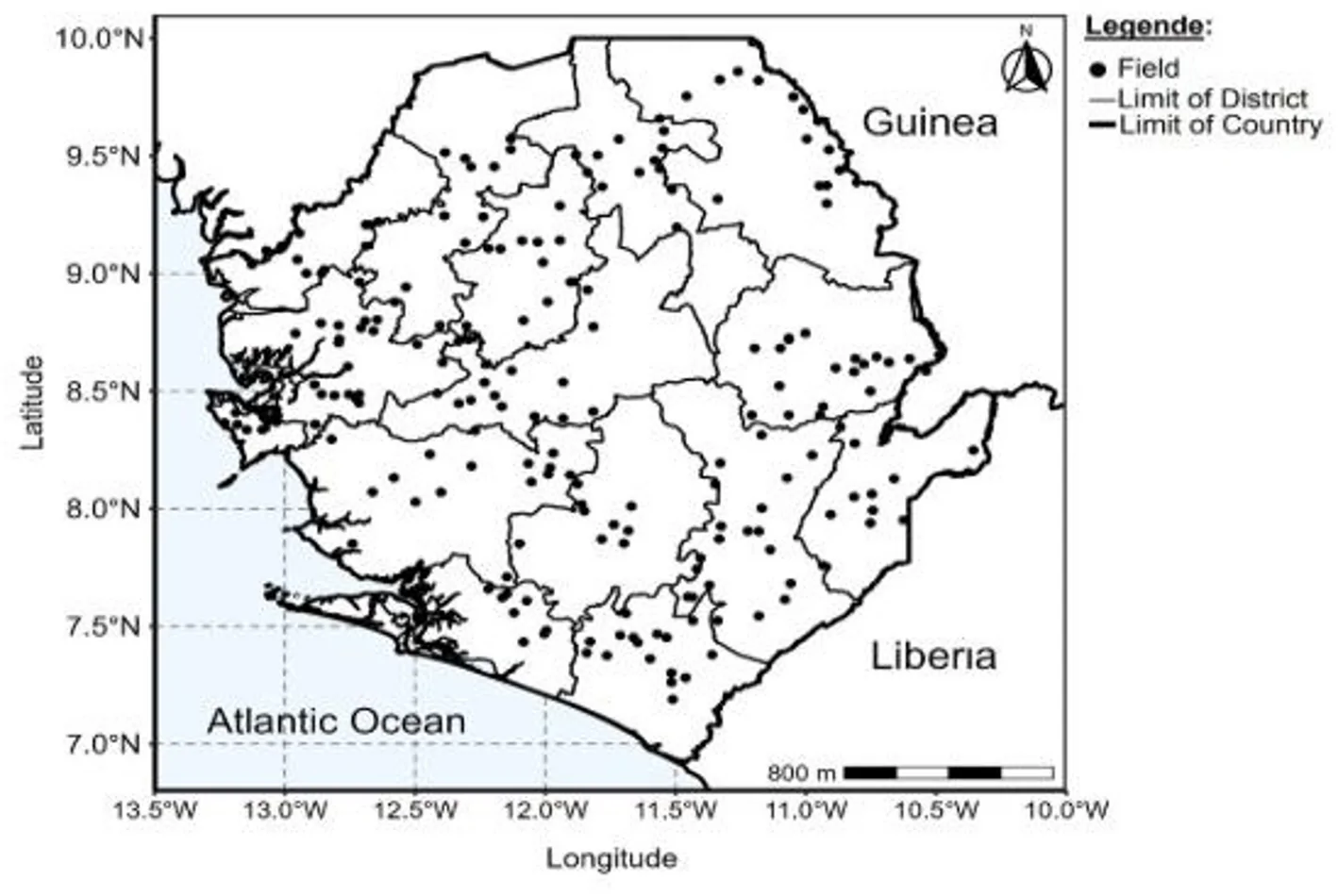
Map of Sierra Leone showing the locations of the 276 farms surveyed in the study

Figure 1. presents the map of Sierra Leone revealing the locations of the 276 farms where the surveys were conducted in 2022. The map was developed using QGIS software v. 2.18.26 (https://qgis.org/downloads/).

### Sample size and research design

A total of 276 farms were surveyed in January 2022 to assess and characterize key agronomic traits of cassava morphological types on farmers’ fields in all regions of Sierra Leone. Of the 276 farms, 18 farms were surveyed per district (i.e. 14 districts accounted for 252 farms), whereas the western region (comprising western rural and western urban districts) accounted for 7 farms per district surveyed. This is because cassava cultivation is more concentrated in the provinces relative production in the western region. The cassava morphological types on farmers’ fields assessed were of different age categories: 1–3, 4–6 and 7–9 months old at the time of the survey. The cassava fields were 10 km apart. At each farm, 30 cassava plants were randomly selected and evaluated along two diagonals using the Central and West African Virus Epidemiology (WAVE) harmonized sampling and diagnostic protocols [8].

### Data collection

Agro-morphological traits comprising both quantitative (number of lobes) and qualitative (apical leaf color, color of leaf vein, prominence of foliar scar, petiole color, leaf color) traits were evaluated based on the agro-morphological descriptor of cassava described by Fukuda et al. [9]. Agronomic, pest and disease data were collected electronically using standardized survey forms and the geolocation coordinates of each field were recorded using a global positioning device (Garmin Dakota TM10 Garmin Limited, St. Olathe, KS, USA). Cameras were also utilized for data collection.

**Fig. 2:**
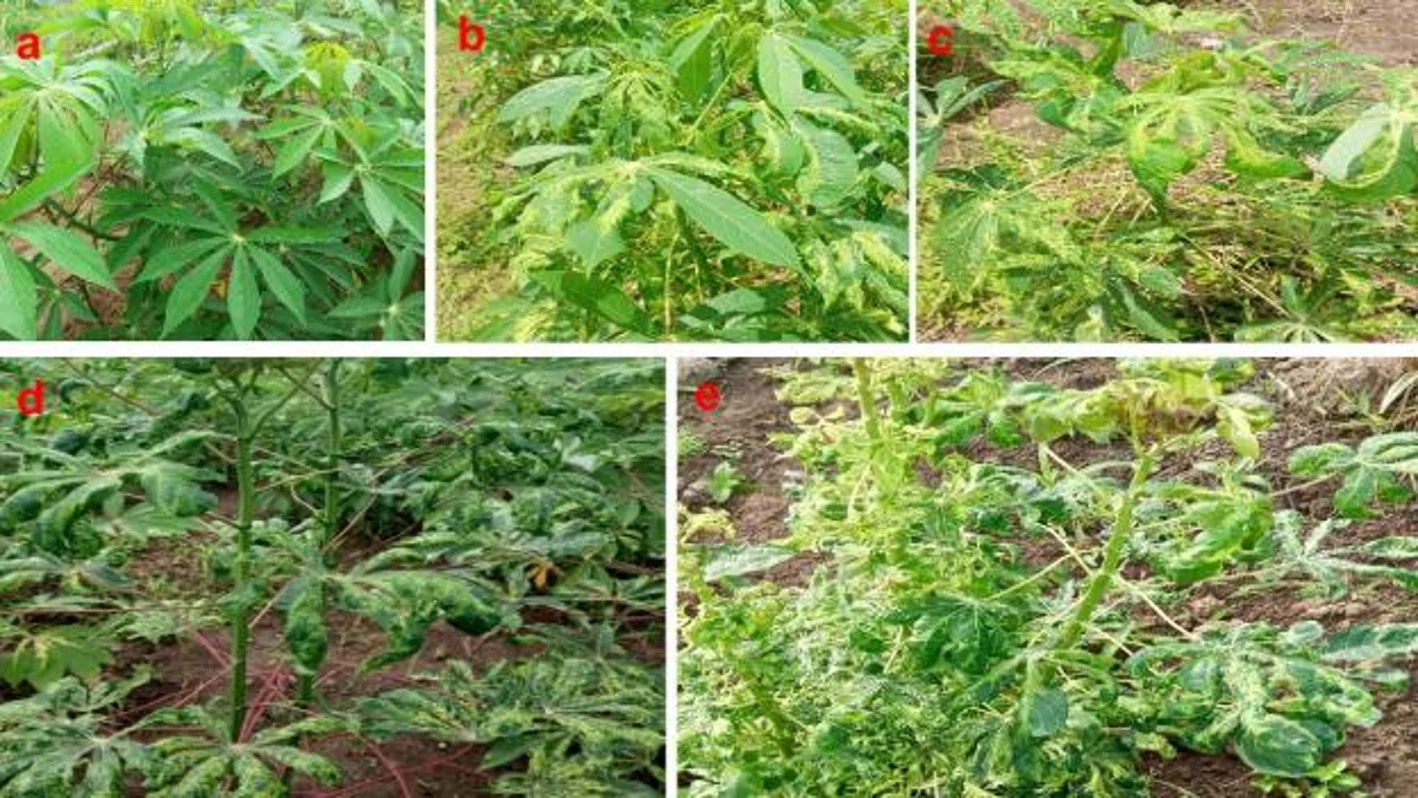
Cassava showing** a** a healthy-looking plant with no visible symptoms of cassava mosaic disease (CMD) on the leaves;** b**–**e** visible symptoms of CMD infection on plant leaves

The severity of cassava leaf symptoms of cassava mosaic disease (CMD) was scored using a scale of 1 to 5, as described by Sseruwagi et al. [10]. Where 1 = no visible symptoms on the leaves (Fig. 2a); 2 = appearance of slight chlorotic spots on the leaves (Fig. 2b); 3 = presence of chlorotic patches on almost all the leaves of the plant ($\:\frac{2}{3}$ of the leaves of the plant) without deformation of the leaf surface (Fig. 2c); 4 = **c**hlorotic patches on almost all leaves, accompanied by deformation (curling) and reduction in leaf area (Fig. 2d); and 5 = severe chlorosis, twisted leaves, deformed and practically reduced to the veins, reduced vegetative apparatus (Fig. 2e).

Whitefly abundance was assessed by counting of five apical leaves per plant. This was achieved by carefully holding the sheet by the petiole between thumb and forefinger, and then gently inverting it so that the adults present on the lower surface are counted. The source or mode of infection of CMD was determined according to Sseruwagi et al. [11]. Plant exhibiting symptoms on the lower and oldest leaves, together with a uniform distribution of symptoms throughout the canopy were classified as cutting-borne infection according to the established CMD diagnostics criteria. In contrast, infection appearing initially on the upper leaves were classified as whitefly-borne. The recorded data were uploaded to iForm’s cloud-based database and integrated into the WAVE Cube prior to analysis.

### Data analysis

Spatial distribution maps were generated using the *geom* function and ggplot2 package in R software, with spatial mapping support from (https://r-charts.com/spatial/maps-ggplot2/) [12]. Categorical data were standardized using the scale() function [13]. Pest populations (whiteflies and grasshoppers), CMD severity, infection mode, farm size, and plant age were analyzed using one-way ANOVA in RStudio.

Pairwise relationships between whitefly abundance, grasshopper abundance, CMD severity, and cassava morphological traits were assessed using Pearson correlation analysis in R [14]. Correlation significance was interpreted following Ratner [15], and results were displayed using correlograms. Correlation coefficients indicated direction and strength of associations, where values of 0, + 1, and − 1 represent no, perfect positive, and perfect negative relationships, respectively. Strength was classified as low (0–0.30), moderate (0.31–0.70), and strong (0.71–1.00) positive or negative relationships [16], with significance set at *P* < 0.05.

## Results

**Fig. 3:**
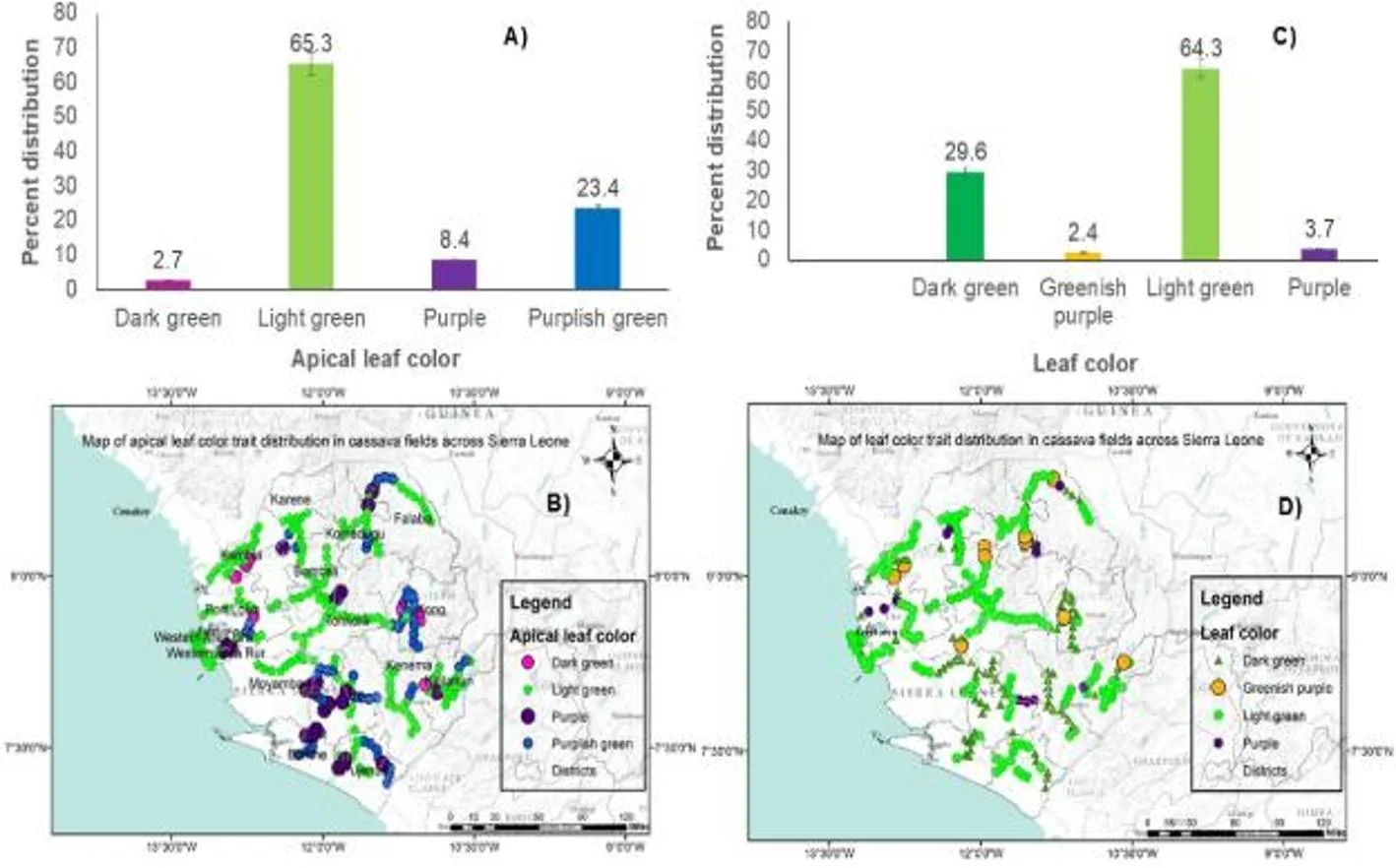
Leaf color traits showing b percent distribution of apical leaf color;** b** map of apical leaf color distribution;** c** percent distribution of leaf color;** d** map of leaf color distribution of cassava morphological types across Sierra Leone

The percent distribution of apical leaf color and map of apical leaf color distribution showed that majority of cassava morphological types possess green apical leaves and are widely spread across all regions of Sierra Leone (Fig. 3A and B). This was followed by morphological types with purplish green apical leaves which are found in all regions, except the western region. Cassava morphological types with purple apical leaves were the third highest, mostly found in the southern region with few in the western and eastern regions. The dark green apical leaves are the lowest cassava morphological types in the country, mostly found in the northwest and eastern regions. Morphological types with light green leaves are widely distributed, followed by those with dark green leaves (Fig. 3C and D). Morphological types with greenish purple leaves were found mostly in the eastern, southern, northern and northwest, while morphological types with purple leaves exhibited the lowest percentage, but are widely distributed in all regions of the country.

**Fig. 4:**
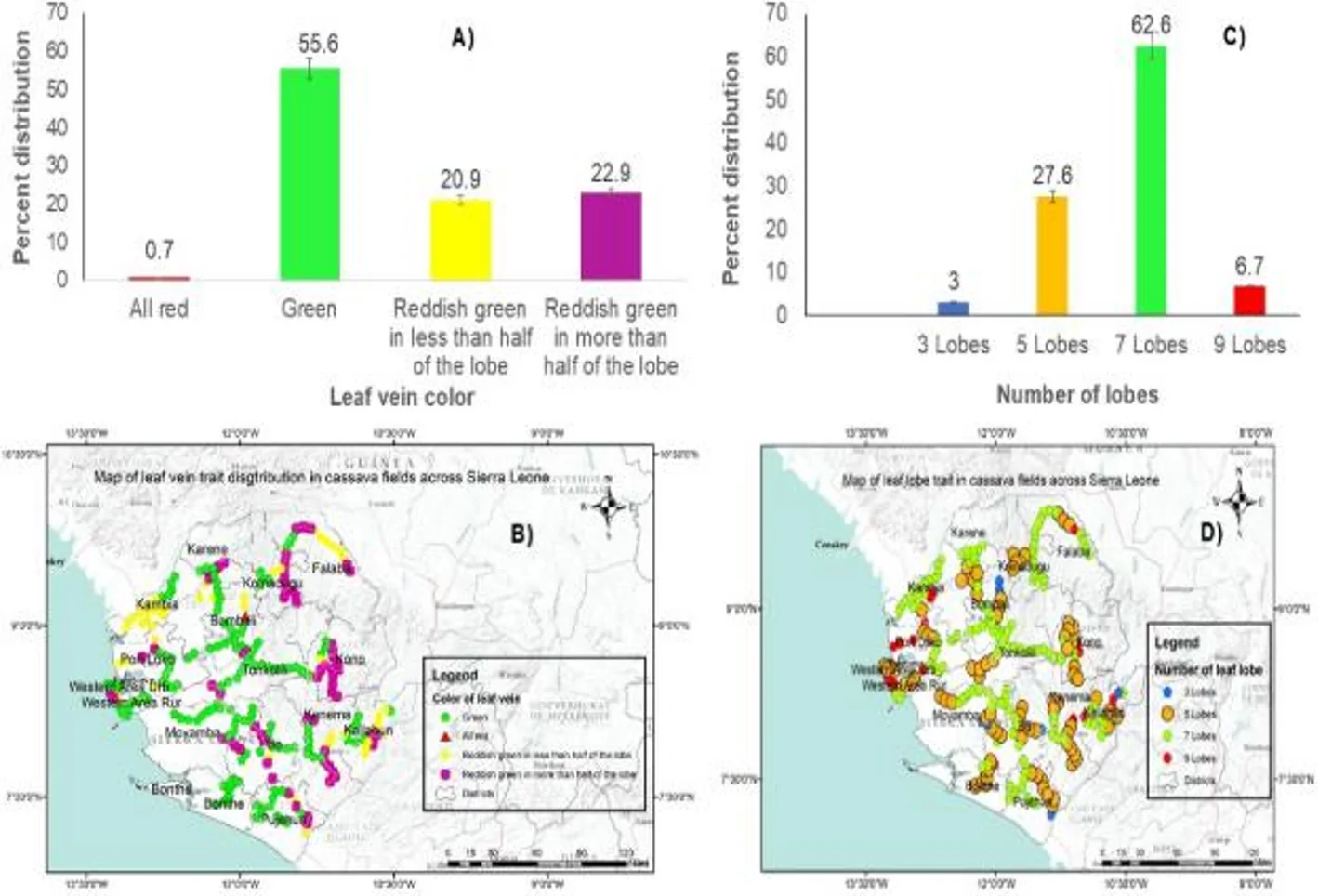
Leaf traits showing** a** percent distribution of leaf vein color;** b** map of leaf vein color distribution;** c** percent distribution of number of lobes;** d** map of number of lobes of cassava morphological types across Sierra Leone

**Fig. 5:**
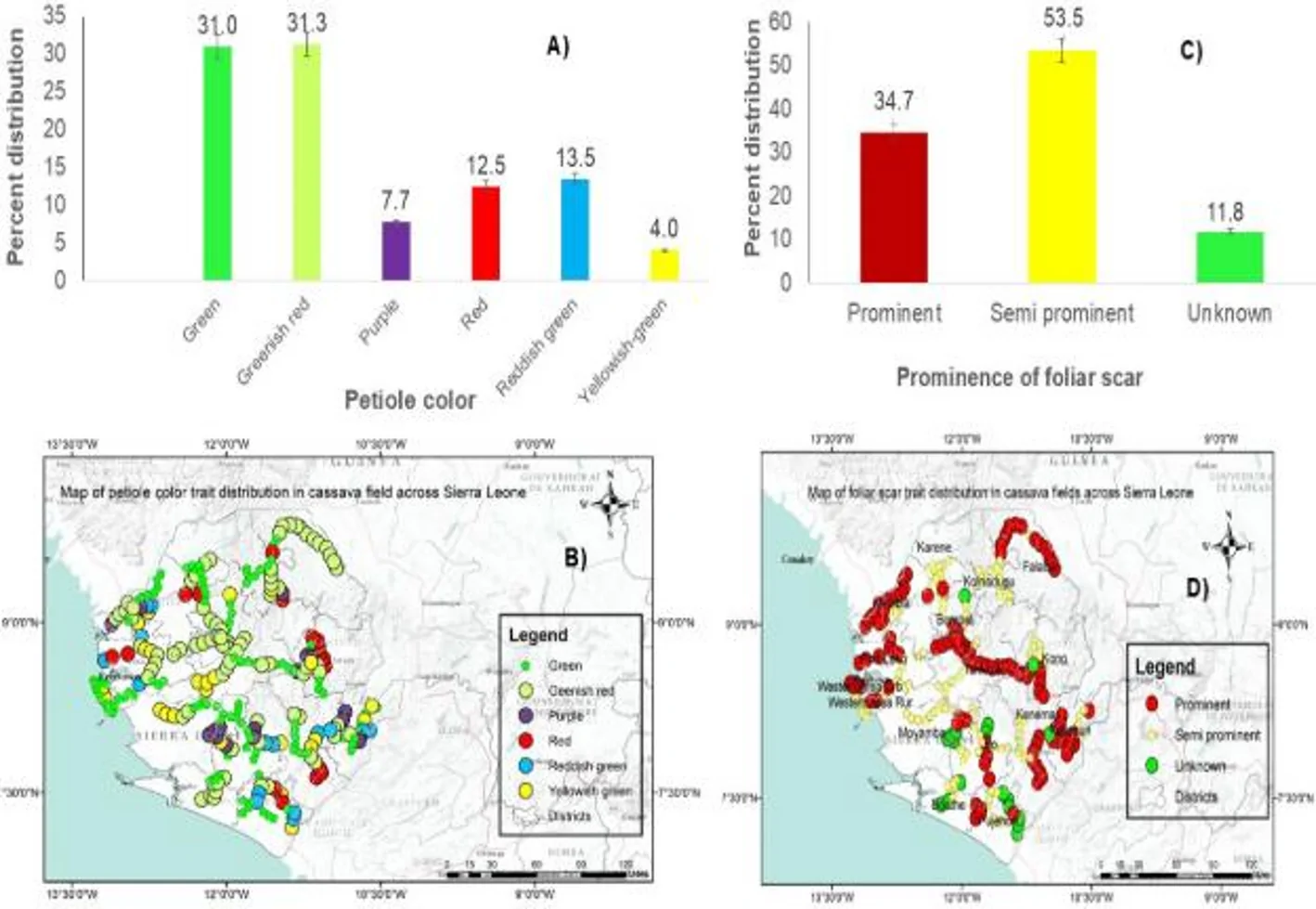
Petiole and foliar scar traits showing** a** percent distribution of petiole color;** b** map of petiole color distribution;** c** percent distribution of prominence of foliar scar;** d** map of prominence of foliar scar of cassava morphological types across Sierra Leone

The percent distribution of leaf vein color and map of leaf vein color distribution showed that majority of the cassava morphological types exhibited green leaf vein widely spread across all the regions, followed by those with reddish in less than half of the lobe and reddish in more than half of the lobe (Fig. 4A, B). Morphological types with all red leaf vein were few and only found in the eastern region. The percent distribution of number of leaf lobes and map of number of leaf lobes showed that morphological types with 7 leaf lobes were the highest (62.6%), followed by those with 5 leaf lobes (27.6%), widely spread across the country (Fig. 4C, D). Morphological types with 3 leaf lobes were found mostly in the southern region and few in northern region, while those with 9 leaf lobes were in the minority, found in all regions. The percent distribution of petiole color and map of petiole color distribution showed that morphological types with green petiole are the most dominant and widely spread across all regions of Sierra Leone, followed by those with greenish red petiole (Fig. 5A, B). Yellowish green and purple petiole morphological types are few, but are widely distributed in all regions of the country. Morphological types with red petiole are found mostly in the eastern, southern and northern regions (Fig. 5B). The percent distribution of prominence of foliar scar and map of prominence of foliar scar showed that about 53.5% of the morphological types exhibited prominent foliar scars, 34.7% had semi prominent foliar scar, while 11.8% of the morphological types were unknown (Fig. 5C, D).

**Fig. 6:**
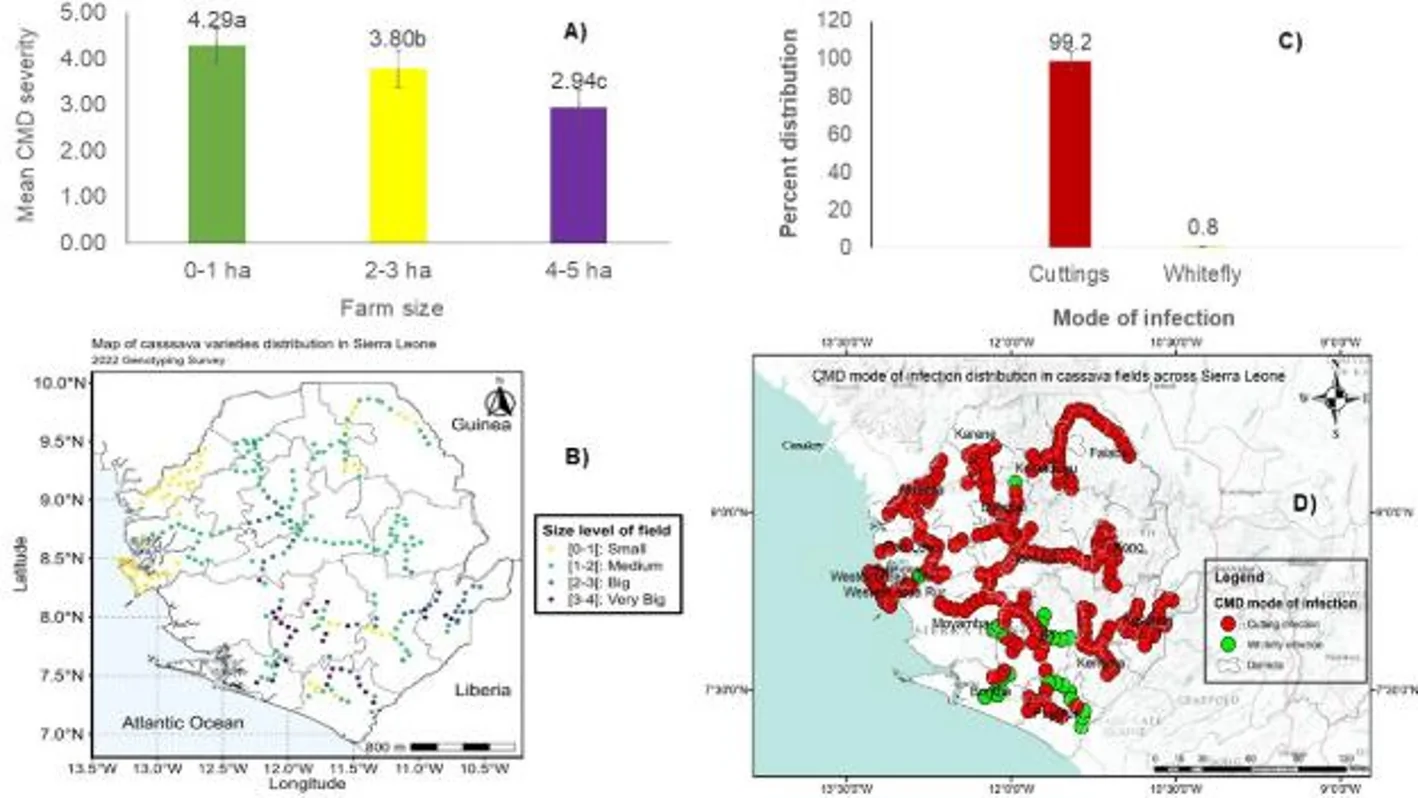
Disease pattern showing** a** severity of cassava mosaic disease (CMD) versus farm size;** b** map of farm size distribution;** c** percent distribution versus mode of CMD infection;** d** map of mode of CMD infection of cassava morphological types across Sierra Leone

Mean CMD severity scores significantly (*p* ≤ 0.05) varied with farm size (Fig. 6A). High CMD severity (4.29) was noted in small farms (0–1 ha), followed by medium farms (2–3 ha) and big farms (4–5 ha) with severity of 3.8 and 2.94, respectively. Majority of the farms are medium size, followed by small farms, which were mostly found in the western region and few in the southern and northern regions (Fig. 6B). Big and bigger farms were mostly found in the southern, eastern and northern regions.

The percent distribution of mode of CMD infection and the map of CMD distribution showed that the main source of the disease infection is through contaminated planting materials, which are widely spread across all regions of Sierra Leone (Fig. 6C, D). It accounted for approximately 99.2% of diseased plants. Infection of the remaining diseased plants (0.8%) may have been caused by the insect vector *B. tabaci*. Few CMD infection was detected in the southern region where tolerant CMD cassava morphological types are cultivated. However, some healthy morphological types such as Tapioka were also found in some communities of the northwest region.

Cassava mosaic disease (CMD) severity ranged from 2.33 to 3.40 across regions and showed no significant differences (F = 2.86, *p* = 0.070), indicating relatively uniform disease pressure. In contrast, whitefly abundance varied significantly (F = 11.04, *p* = 0.001), with the highest population in the western region (7.00 ± 1.58) and the lowest in the eastern region (3.03 ± 1.58); Tukey results show western differed from all other regions. Grasshopper populations also differed significantly (F = 54.18, *p* < 0.001), being highest in the eastern and southern regions and lowest in the western region (Table 1). The results reveal clear spatial heterogeneity in pest distribution, while CMD severity remains relatively stable across regions, indicating that pest dynamics are more region-specific than disease expression in cassava fields.

**Table 1:** Regional variation in cassava mosaic disease (CMD) severity, whitefly abundance, and grasshopper populations recorded on cassava farms across Sierra Leone

Region	CMD severity per plant (Mean ± SD)	Whiteflies per plant (Mean ± SD)	Grasshoppers per plant (Mean ± SD)
Eastern	3.02 ± 0.38	3.03 ± 1.58a	12.00 ± 4.16a
Southern	2.33 ± 0.38	4.01 ± 1.58a	10.10 ± 4.16a
Northern	3.40 ± 0.38	5.12 ± 1.58a	5.11 ± 4.16b
Northwest	3.10 ± 0.38	5.23 ± 1.58a	5.34 ± 4.16b
Western	3.00 ± 0.38	7.00 ± 1.58b	2.00 ± 4.16b
Overall Mean ± SD	2.97 ± 0.38	4.88 ± 1.58	6.91 ± 4.16
Variable	df (between, within)	F-value	*p*-value
CMD severity	(4, 271)	2.86	0.070 ns
Whitefly abundance	(4, 271)	11.04	0.001 **
Grasshopper abundance	(4, 271)	54.18	< 0.001 ***

Source: Survey data, 2022

*Means followed by different letters within a column differ significantly according to Tukey’s HSD test at *P* < 0.05. No mean separation was performed for variables with non-significant ANOVA results (*P* > 0.05). Analysis was based on farm-level observations (i.e. mean of 30 plants per farm)

**Fig. 7:**
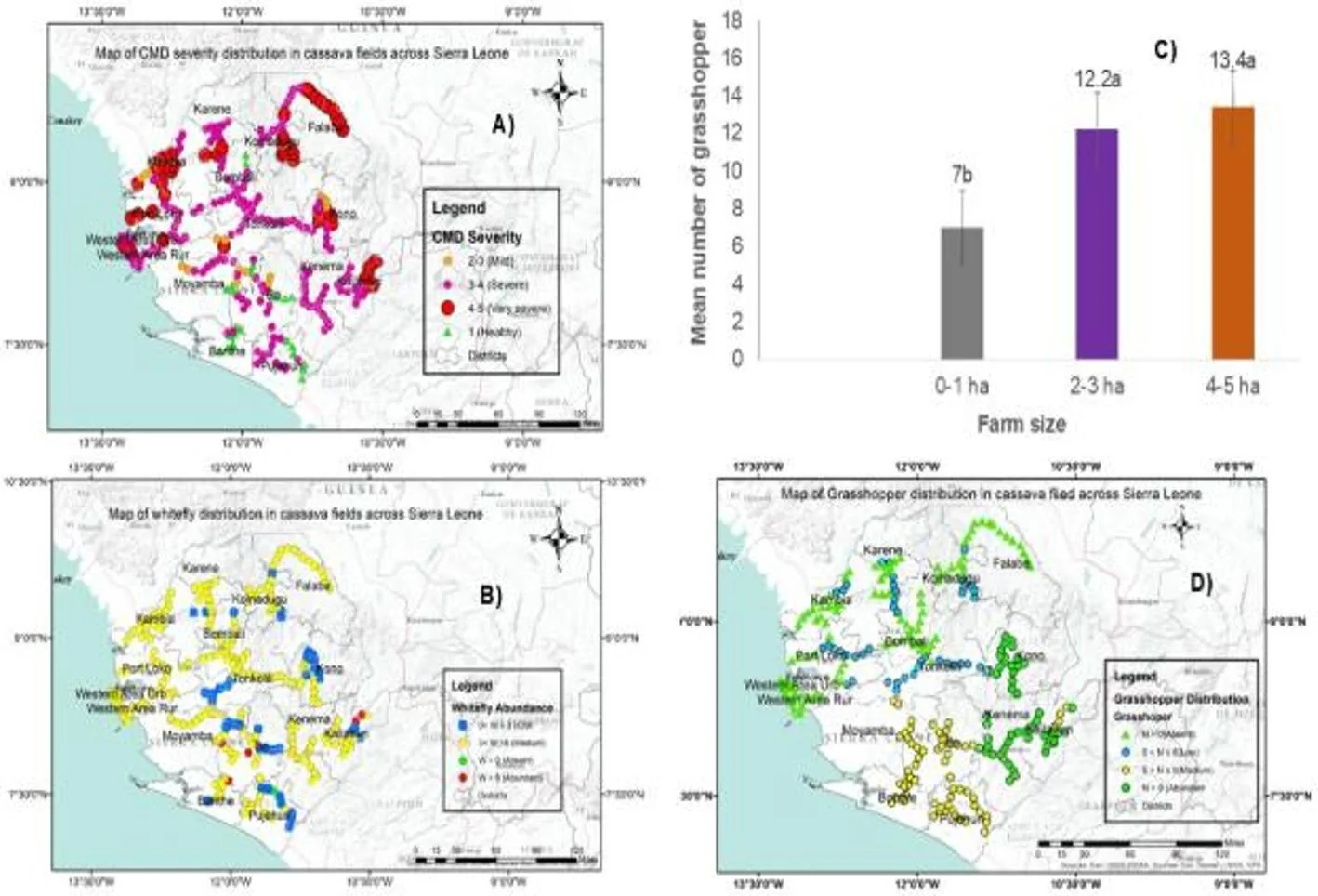
Disease pattern showing** a** map of distribution of cassava mosaic disease (CMD) severity;** b** map of distribution of whitefly in farmers’ field;** c** mean grasshopper population by farm;** d** map of distribution of grasshopper in cassava farms across Sierra Leone

Map of severity of CMD distribution showed wide spread infection of the disease across the country (Fig. 7A). Morphological types with very severe CMD symptoms were found in the western, northern, northwest, and eastern regions. Morphological types with mild CMD symptom were found in the southern, eastern and northwest regions, while few CMD tolerant morphological types were found only in the southern and northwest regions. Majority of the farms had 3–6 whiteflies per plant, mostly found in the western, northern and northwest regions with few farms in southern and eastern regions (Fig. 7B). Farms that had low whitefly population (0–3) were found in the southern, eastern and northern regions. Farms with more than 6 whiteflies were found in the southern and eastern regions, whereas few farms with no visible whiteflies were found in the southern region. Mean number of grasshoppers significantly (*p* < 0.05) varied with farm size (Fig. 7C). The highest number of grasshoppers (13.4) was observed on the big farms (4–5 ha), followed by the medium farms (3–4 ha), which recorded 12.2 grasshoppers per plant, and the lowest of 7.0 grasshoppers was observed on small farms (0–1 ha). Map of distribution of grasshopper in cassava farms across Sierra Leone is presented in Fig. 7D. All farms in the eastern region had more than nine grasshoppers, followed by southern region with 5–9 grasshoppers. Majority of the farms in the northern and northwest regions had low grasshopper population ranging from 0 to 5.

**Fig. 8:**
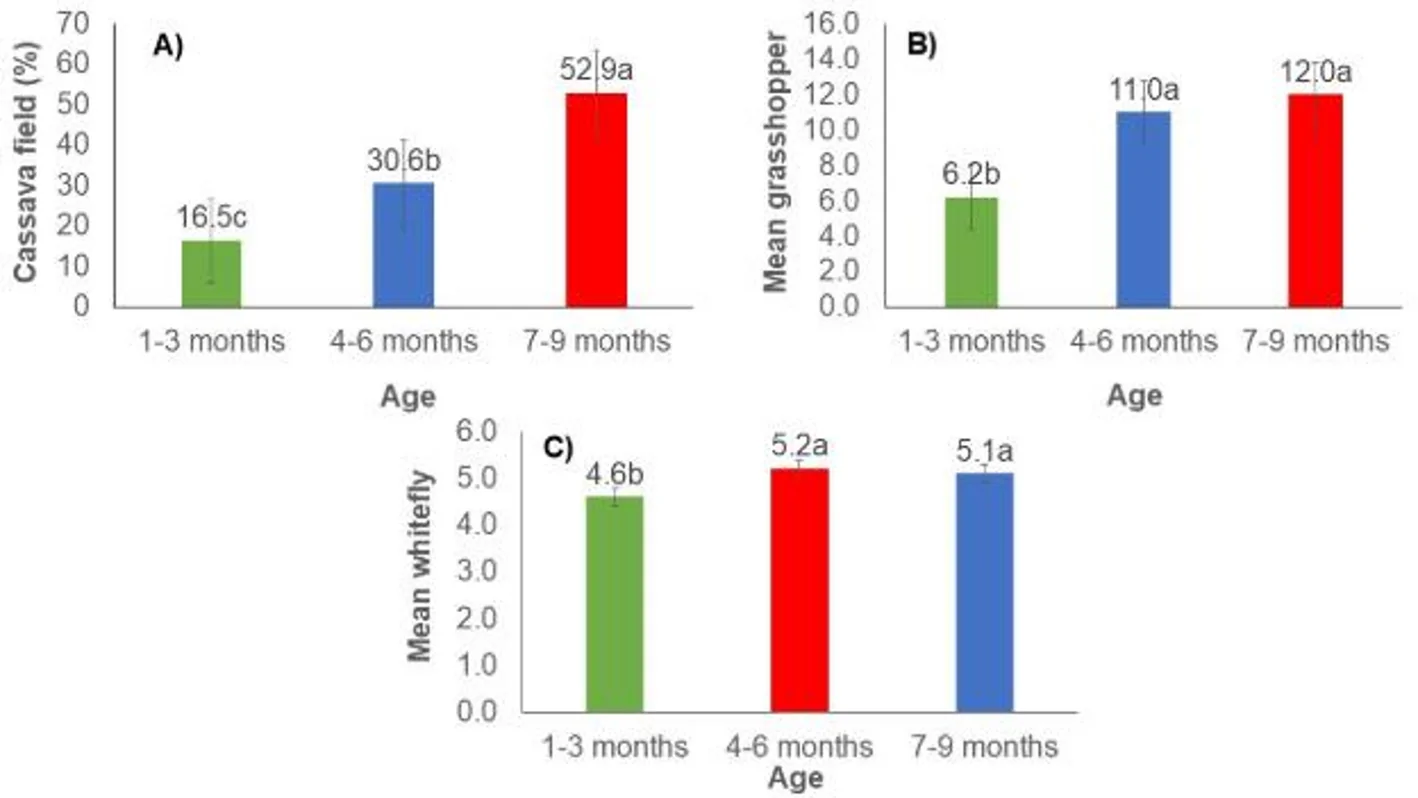
Cassava farms showing** a** percent farms per age category;** b** mean grasshopper population by age; and (c) mean whitefly abundance per age category of plants sampled across Sierra Leone

Figure 8 presents cassava farms showing percent of farms assessed per age category, mean grasshopper population per age category and mean whitefly abundance per age category. Accordingly, whiteflies and grasshoppers were higher in cassava plants that were 4–6 and 7–9 months old than the 1–3 months old plants.

**Fig. 9:**
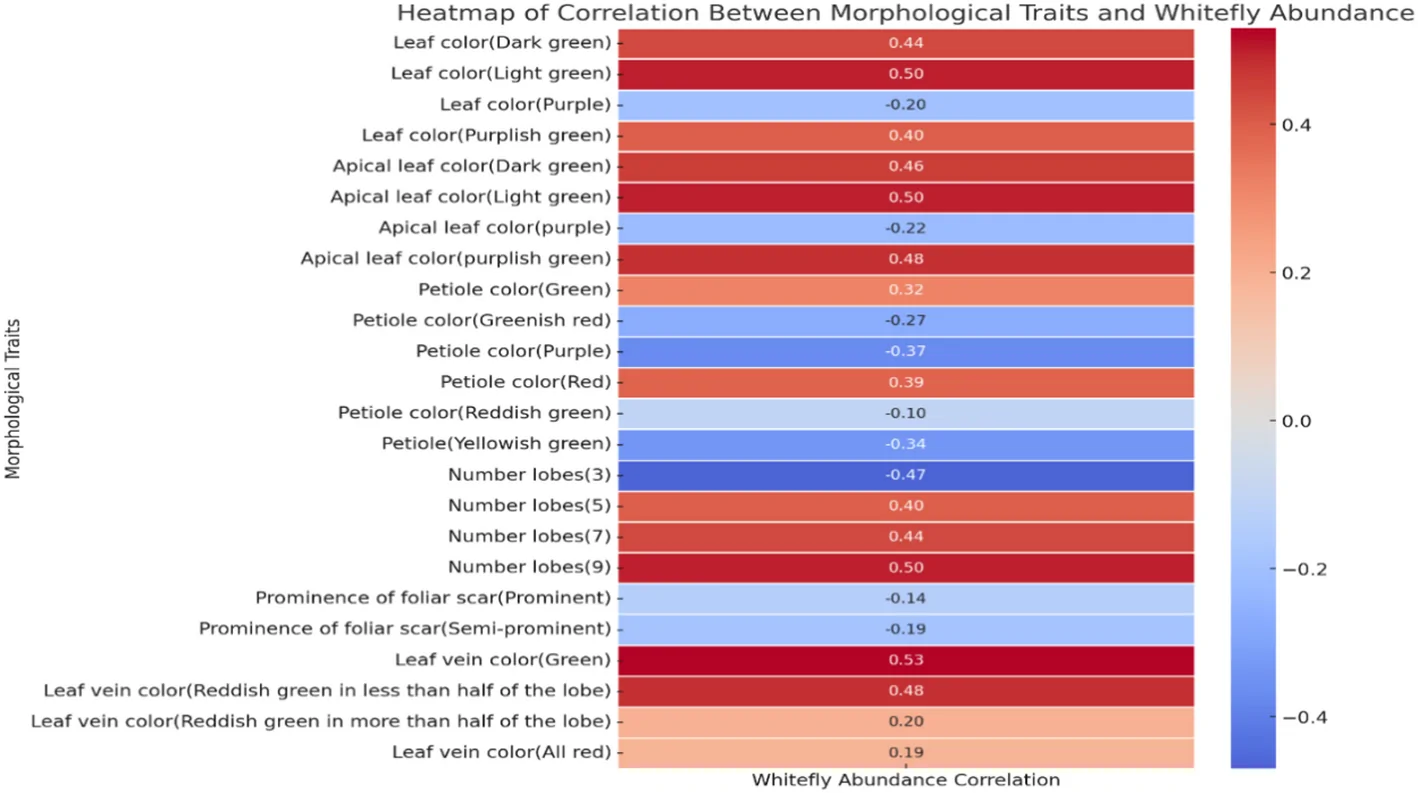
Pearson correlation heatmap illustrating associations between cassava morphological traits and whitefly abundance. *Positive correlations are represented by warmer colors and negative correlations by cooler colors, with color intensity indicating correlation strength

The correlation analysis revealed distinct associations between cassava morphological characteristics and whitefly abundance (Fig. 9). Moderate positive correlations were observed between whitefly abundance and light green leaf color (*r* = 0.50), between whitefly abundance and light green apical leaf color (*r* = 0.50), between whitefly abundance and purplish-green apical leaves (*r* = 0.48), between whitefly abundance and dark green leaves (*r* = 0.44), between whitefly abundance and seven-lobed leaves (*r* = 0.44), and between whitefly abundance and nine-lobed leaves (*r* = 0.50). These findings suggest that whiteflies may preferentially colonize morphological types possessing larger photosynthetically active leaf surfaces and greener foliage, which are often associated with higher nitrogen content and greater nutritional suitability for sap-feeding insects. Conversely, negative associations were observed between whitefly abundance and purple petiole color (*r* = – 0.37), between whitefly abundance and yellowish-green petiole color (*r* = -0.34), and between whitefly abundance and three-lobed leaves (*r* = – 0.47). This association may be related to purple pigmentation in cassava tissues caused by elevated concentrations of anthocyanins and other phenolic compounds, which may have contributed to reduced insect feeding and oviposition in several plant species. The negative association with fewer leaf lobes may be also related to the reduced canopy complexity and lower attractiveness for whitefly colonization. However, further trials are imperative to determine whether there is a functional relationship between reduced canopy complexity and lower attractiveness for whitefly colonization; between pigmentation and nutritional suitability for sap-feeding insects; and whether such relationships are influenced by morphological types-specific characteristics and environmental factors.

**Fig. 10:**
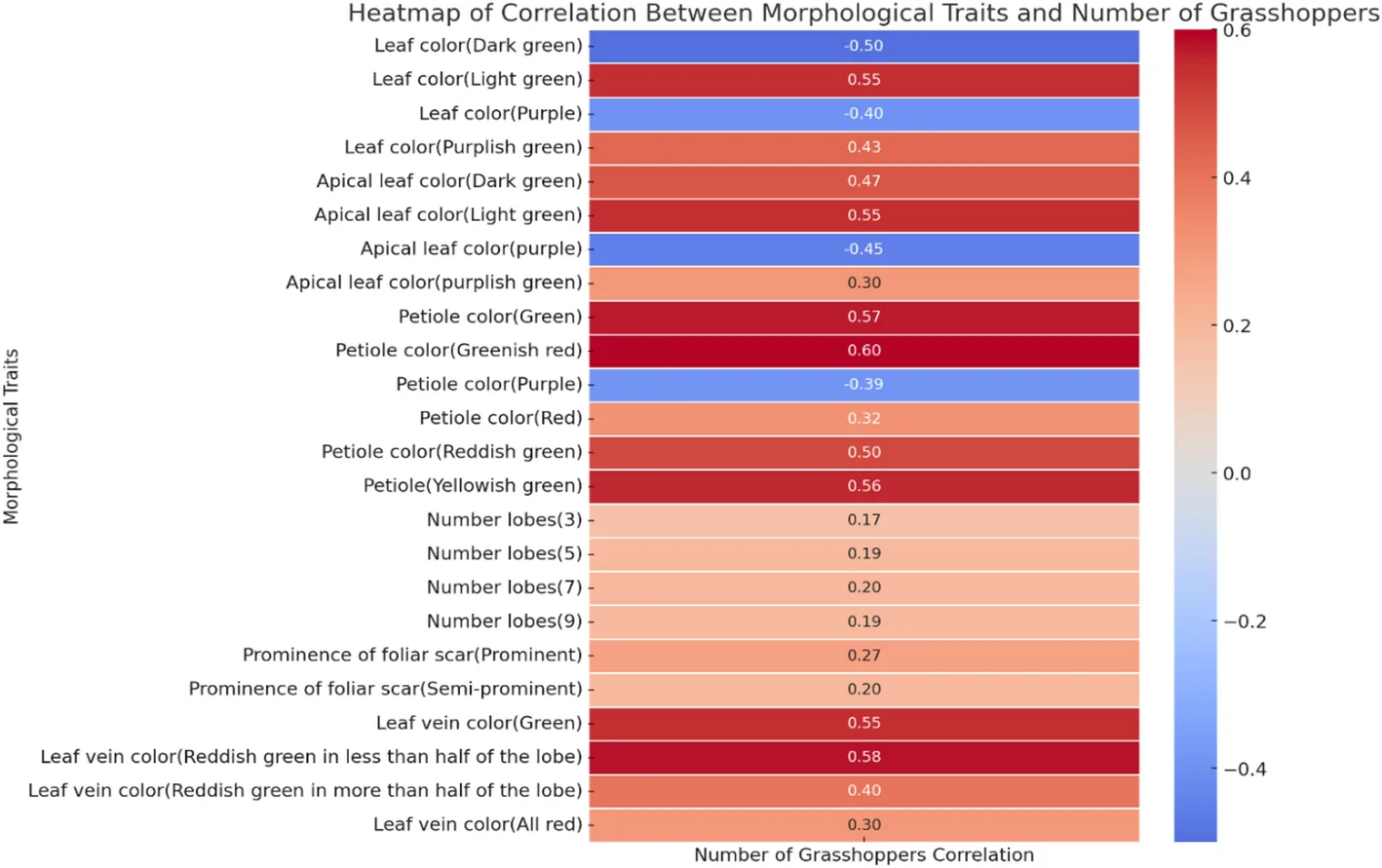
Pearson correlation heatmap showing relationships between cassava morphological descriptors and grasshopper abundance across surveyed farms in Sierra Leone

The correlation heatmap revealed that grasshopper abundance was associated with several cassava morphological traits (Fig. 10). Moderate positive correlations were observed with light green leaf color (*r* = 0.55), green leaf veins (*r* = 0.55), reddish-green leaf veins in less than half of the lobe (*r* = 0.58), green petioles (*r* = 0.57), greenish-red petioles (*r* = 0.60), and yellowish-green petioles (*r* = 0.56). These associations suggest that grasshoppers may preferentially exploit morphological types exhibiting vigorous vegetative growth and greater foliar biomass, thereby providing increased feeding opportunities. In contrast, moderate negative correlations were observed between grasshopper abundance and purple leaf color (*r* = – 0.40), purple apical leaves (*r* = – 0.45), and purple petioles (*r* = – 0.39). One possible explanation is that anthocyanin-rich tissues may have possible linkage to anti-herbivory responses that affect many plant species. The observed trend indicates that pigmentation-related traits could serve as useful phenotypic indicators during preliminary screening for reduced susceptibility to chewing insect pests. Nevertheless, further experimental studies are required to determine whether these morphological characteristics directly influence grasshopper feeding behavior.

**Fig. 11:**
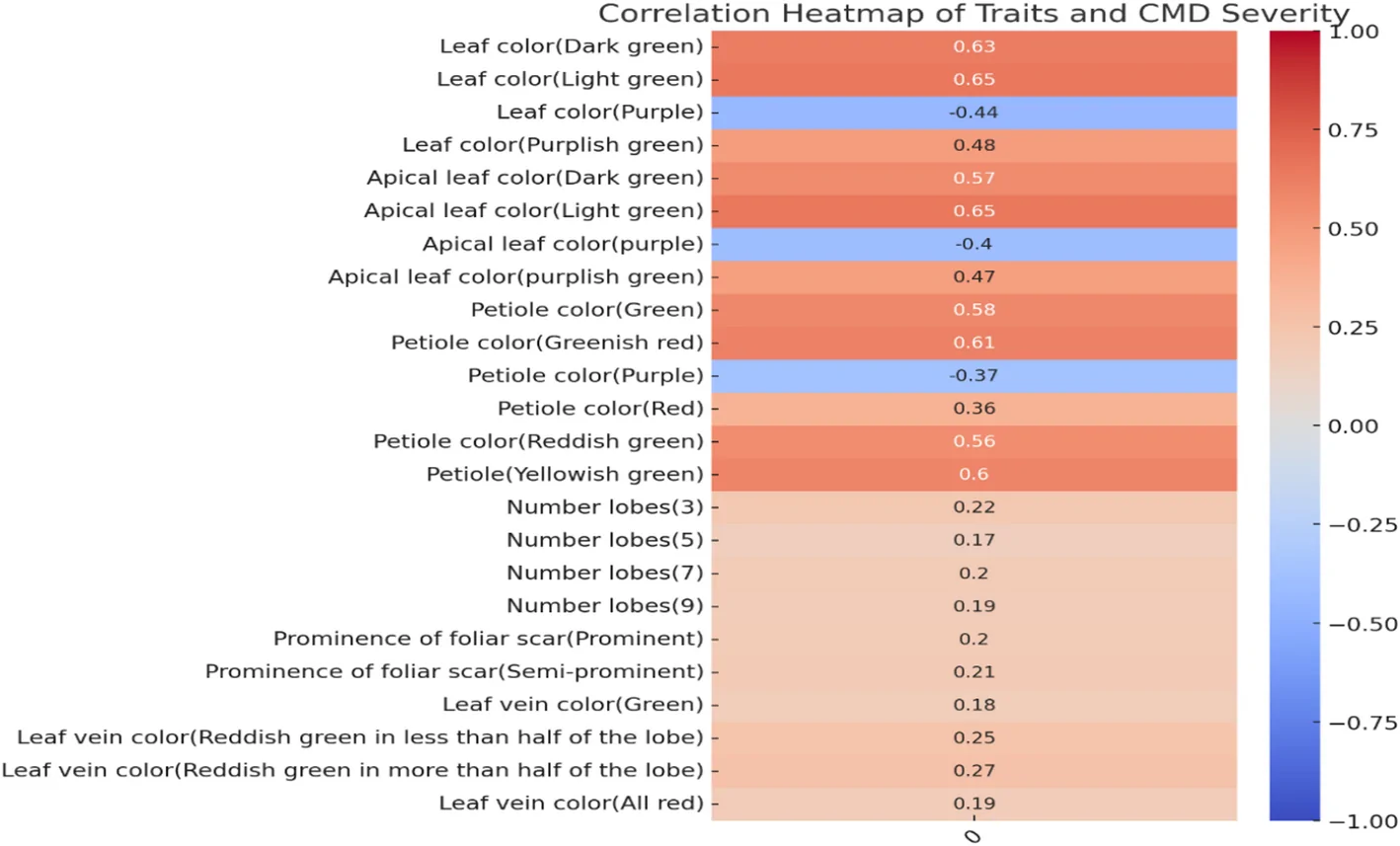
Pearson correlation heatmap depicting associations between cassava morphological traits and CMD severity scores across surveyed cassava fields

The association between CMD severity and morphological traits revealed several noteworthy patterns (Fig. 11). Moderate positive correlations were observed between CMD severity and light green leaves (*r* = 0.65), between CMD severity and light green apical leaves (*r* = 0.65), between CMD severity and dark green leaves (*r* = 0.63), between CMD severity and greenish-red petioles (*r* = 0.61), between CMD severity and yellowish-green petioles (*r* = 0.60), and between CMD severity and green petioles (*r* = 0.58). These findings suggest that certain morphological traits may have co-occurred with increased disease severity within the surveyed cassava populations. These traits are commonly associated with local varieties, which are more susceptible to the CMD.

Negative correlations were observed between CMD severity and purple leaf color (*r* = − 0.44), between CMD severity and purple apical leaves (*r* = – 0.40), and between CMD severity and purple petioles (*r* = – 0.37). This association may be related to a potential linkage between pigmentation pathways and plant defense responses. Anthocyanins and related phenolic compounds are known to play a crucial role in stress tolerance and pathogen defense mechanisms in different crop species. However, further trials are imperative to determine whether anthocyanins and related phenolic compounds play a crucial role in CMD stress tolerance and pathogen defense in cassava.

## Discussion

The distribution maps revealed wide variation in morphological traits possibly attributable to influence by cultural practices, environmental conditions, and phenotypic variation. Cultural practices play key role in the distribution of morphological traits as farmers select and name cassava morphological types based on morphology (e.g. tree), food (e.g. Cooksoon), social and economic interest (e.g. IDA), village or community (e.g. Warima) or who they got the variety from (e.g. Ya Kanu) [17, 18]. Moreover, the diverse cultural uses of cassava as food, feed, medicinal and other traditional applications influence the distribution of morphological traits in cassava growing regions of Sierra Leone [19]. The widespread occurrence of several morphological traits across regions suggests the combined influence of phenotypic diversity, farmer-mediated selection, and local environmental adaptation. Farmers often retain and exchange planting materials from morphological types that exhibit desirable agronomic characteristics, thereby promoting the persistence and dissemination of specific traits. Consequently, characteristics such as cassava mosaic disease, green apical leaves, green leaf veins, and seven-lobed leaves were commonly observed across the study regions. The continued propagation of these preferred morphological types through informal seed systems has likely contributed to the maintenance of these traits across diverse agroecological conditions. Previous studies show that leaf color and leaf shape are key traits farmers use to identify and select cassava varieties, making them important in local seed systems [2, 19]. Similar patterns have also been reported in other cassava-growing regions, where morphological traits strongly reflect farmer preferences and variety recognition systems [20].

The spread of similar traits across distant regions is also explained by the frequent exchange of stem cuttings between farmers, which allows planting materials to move widely across communities [21]. In addition, occasional recruitment of volunteer seedlings adds new variation into farmer fields, further enriching diversity [22]. The distribution of morphological traits of cassava is also influenced by cultural practices such as planting density [23] and the use of organic and inorganic fertilizers [24]. The observed morphological diversity provides valuable germplasm resources for cassava improvement programs. Traits exhibiting wide spatial distribution may represent stable characteristics that can be exploited in breeding programs aimed at improving adaptation, yield stability, and resistance to pests and diseases. The identification of regions harboring distinct trait combinations also provides opportunities for targeted germplasm collection and parental selection and the pattern observed from the study suggests that cassava diversity in Sierra Leone is shaped more by farmer practices and seed movement than by geography alone, with local conditions only fine-tuning which traits persist.

Field symptoms based on established epidemiological studies have confirmed the presence and East African Cassava Mosaic Virus and the Ugandan variant. Mixed infection has also been detected in several fields in Sierra Leone. This is consistent with Alabi et al. [25] who found that cassava mosaic disease was mainly disseminated through contaminated cuttings. Although mean CMD severity varied numerically among regions, the differences were not statistically significant. The low level of CMD may be attributable to the higher adoption of improved cassava varieties by farmers in the southern region. This result is in disagreement with the findings of Samura et al. [26] who reported that the highest CMD severity (2.67) was found in the rain forest ecology (eastern region). The variance is partly attributable to the varying planting dates, morphological types, ecology and climate that support whitefly population, disease infectivity and development. In this survey, the adult whitefly and grasshopper numbers per plant averaged 4.88 and 6.91, respectively. These values are higher than those recently reported for Burkina Faso [27], but lower (5.57) than those reported by Doungous et al. [28]. At the regional level, whitefly counts were higher in the west region and grasshopper counts were higher in the southern than the other regions. Cassava mosaic disease severity and whitefly population were higher in cassava morphological types within 4–6 months old, whilst grasshopper population was higher in morphological types that were 7–9 months old. These findings are consistent with Soro et al. [27] who reported that cassava mosaic severity was higher in young cassava fields. The spatial variation in whitefly and grasshopper abundance in the present study suggests that pest pressure is not uniform across cassava-growing regions. Despite the relatively low number of whiteflies the numbers are sufficient to enhance vector borne transmission as whitefly population continues to rise amidst changing climatic conditions.

The CMD symptoms in most of the cassava fields were cutting borne compared to those caused by whiteflies, indicating a widespread use of local susceptible varieties. Farms with no visible whiteflies were found in the southern region, while those with high whitefly abundance were detected in the western region and some farms in the southern region. This could be related to the high relative humidity and low altitude of these regions as they are close to the Atlantic Ocean. Findings also indicate that whitefly counts decreased with increasing altitude, as also reported in Madagascar and Tanzania by Harimalala et al. [29] and Szyniszewska [30], respectively. From the visual assessment on the level of CMD severity, it was revealed that farms with severe CMD symptoms were found in every region of the country, indicating that CMD symptom is widely distributed across the country. Few farms with mild CMD symptom were found in the southern, eastern, northern and northwest regions suggesting the wide use of improved morphological types and or phytosanitation in these regions. Samura et al. [26] reported low CMD severity of 1.26 in Bonthe district of the southern region. This district is noted for increased use of resistant morphological types. Resistant morphological types exhibit lower concentration of CMD than susceptible ones, as demonstrated serologically by Fargette et al. [31]. This suggests that resistant morphological types are poor sources of inoculum from which vectors can acquire and transmit virus to uninfected plants. Thus, the adoption of such morphological types contributes to a decrease in the amount of inoculum present in the cassava farming communities or regions and restrict the spread of CMD to any susceptible morphological types being cultivated. The probable consequence is that stands of virus-resistant morphological types sustain little damage or yield loss due to CMD, even under conditions of high inoculum pressure since only a proportion of the plants are infected and those infected sustain little damage and spread. Moreover, CMD occurs in only a proportion of the cuttings collected from infected plants of resistant morphological types so that the incidence of CMD in the cuttings is usually less than in the stand from which they were collected. It is probable that this ‘cleansing’ effect is further enhanced if infection decreases vegetative vigor, or farmers discriminate in favor of vigorous and/or symptomless plants when selecting cuttings for cultivation. For these reasons the incidence of infection is unlikely to increase progressively in successive cycles of propagation, as might otherwise be expected with a vector-borne virus of a vegetatively-propagated crop. Rogueing is one of the official schemes for the selection and maintenance of CMD-free stocks for release to farmers. However, this technique may lead to a progressive and unacceptable decrease in stand in areas of high inoculum pressure where much spread occurs, suggesting that rogueing has little effect in reducing spread within treated fields. Rogueing is probably most effective when practiced by groups of farmers or throughout the whole cassava farming communities. Use of CMD-free stem cuttings as planting material could be imperative since cassava plants, including those affected by CMD, regenerate readily from stems left in or on the ground at harvest. Moreover, farmers often harvest piecemeal and from the most vigorous unaffected plants within a stand and then establish new cuttings in the gaps created. This means that young plants often develop beneath or immediately alongside older infected ones. These are potential sources of virus inoculum and also of other pathogens and pests including the cassava green mite (*Mononychellus tanajoa* Bondar) and the cassava mealybug (*Phenacoccus manihoti* Matile-Ferrero). One of the novel techniques utilized for the rapid generation of CMD-free stem cuttings is the semi autotrophic hydroponics (SAH). The technique utilizes virus indexed plantlets from tissue culture as starter materials. The finding that approximately 99.2% of CMD infections were associated with contaminated planting materials highlights the critical importance of strengthening cassava seed systems in Sierra Leone. Establishment of decentralized multiplication centres, certification of disease-free planting materials, and farmer training on stem selection could substantially reduce CMD dissemination through infected cuttings. Most of the cassava farms in Sierra Leone are medium farms (2–3 ha). Small farms (0–1 ha) are found more in the western region, while few big farms (4–5 ha) are noted in the eastern and northern regions, and more big farms are found in the southern region where cassava farming is done mainly for commercial purposes.

The correlation analysis indicates that cassava morphological types with green leaves, multiple lobes and green leaf were the most dominantly cultivated across the country and tend to attract more whiteflies, whereas those with purple petiole and fewer lobes were seemingly less infested by pest. Morphological types with purple apical leaves were less susceptible to CMD attacks. The less attractiveness of whitefly to these morphological types might be linked to the presence of secondary metabolites like anthocyanins which have insect-repellent properties. These findings indicate that the morphological traits including the leaf attributes play significant roles in morphological types identification and selection by farmers for cultivation, consumption, and leafy vegetable markets. Results are in conformity with the work of Karim et al. [2], who opined that leaf attributes are important for cassava identification and selection for leafy vegetable markets in Sierra Leone, where cassava foliage is widely consumed. The negative association between CMD severity and farm size indicates that the CMD severity increases in small farms where susceptible morphological types, disease inoculum and congenial environmental conditions favor disease growth and development. This supports the view of Samura et al. [26] who opined that CMD severity was low in farms where resistant varieties were planted in Bonthe district.

The GIS or spatial analytical technique is known as a powerful tool utilized in the field of agriculture to revolutionize farming practices and pave the way for a more sustainable and efficient future. However, its wide utilization is limited by cost and accessibility of technology, data quality and integration, and technical expertise and training. The cost of the GIS software and hardware can be expensive, and not all farmers have the financial resources to invest in it. The quality and integration of GIS data are expensive to collect and difficult to integrate with other types of data, such as crop yield data or weather data. The GIS technology can be complex and difficult to use, suggesting that farmers and other users should have a certain level of technical expertise in order to use it effectively. Moreover, farmers, students and researchers need to be trained on how to use GIS technology in order to get the most out of it.

The present study was done for a year and only for cassava. It utilized phenotypic data to explore the existing phenotypic variability for the studied parameters. The limitations of phenotypic data in exploring phenotypic variability and spatial analysis are multifaceted. These limitations highlight the need for improved data collection, management, and analytical techniques to effectively explore phenotypic variability and spatial analysis. Some of the key limitations include: poor algorithm performance, complex traits (i.e. phenotypic data are limited in scale, density and accuracy, making it difficult to quantify complex traits); data availability (i.e. lack of a consensus-based, human- and machine-interpretability of phenotypes and their genomic and environmental contexts); integration constraints (i.e. integration of morphological types and phenotype datasets from heterogeneous sources is cumbersome, thereby limiting their effective utilization for genomic selection and translational research). These limitations highlight the necessity of improved data collection, management, analytical techniques to effectively explore phenotypic variability and spatial analysis. Addressing these constraints are critical for advancing our understanding of plant genetics, improving crop breeding and management. According to Amara et al. [32], utilization of molecular techniques such as SNP genotyping, genotyping-by-sequencing, and marker-assisted selection, would facilitate the identification of resistance loci and predictive breeding. Integration of spatial datasets with genomic information through environmental association analyses would enhance understanding of genotype-environment interactions thereby supporting climate-resilient breeding strategies, disease surveillance, predictive modeling of outbreaks, targeted germplasm deployment, and improved selection efficiency across various production environments. This offers a comprehensive pathway to develop disease-resistant cassava morphological types, thereby strengthening food security and sustainable agriculture in Africa.

## Conclusion

This study establishes the existence of useful phenotypic variability in the regional distribution of agronomic traits that could be exploited by plant breeders, plant scientists, students, lecturers, policymakers, research and development partners. Majority of the cassava morphological types in Sierra Leone exhibit green apical leaf, light green leaf, green leaf vein, and prominent foliar scar with seven lobes. Whiteflies and grasshoppers had higher populations in the western and eastern regions, respectively; whereas few farms in the southern region had no visible whiteflies. The main source of cassava mosaic disease (CMD) infection is through contaminated cuttings. Plants from infected cuttings exhibit moderate infection of CMD across the country. Mean CMD severity depends on farm size, with the small (0–1 ha), medium (2–3 ha) and big (3–4 ha) farms exhibiting high, medium and low CMD infections, respectively. Findings are relevant in making informed decision on genotypic selection for production, conservation, utilization, and genetic improvement of cassava.

## Data Availability

The datasets generated during and/or analyzed during the current study are available from the corresponding author on reasonable request.
